# A novel approach for quantitative assessment of mucosal damage in inflammatory bowel disease

**DOI:** 10.1186/1746-1596-8-156

**Published:** 2013-09-20

**Authors:** Ismail I Matalka, Faruq A Al-Omari, Rola M Salama, Alia H Mohtaseb

**Affiliations:** 1 Department of Pathology and Laboratory Medicine, Jordan University of Science and Technology, Irbid, Jordan; 2 Computer Engineering Department, Hijjawi Faculty for Engineering Technology, Yarmouk University, Irbid 21163, Jordan

**Keywords:** Inflammatory bowel disease, Mucosal damage assessment, Morphometric features, Medical imaging, Artificial intelligence in medicine, Computer assessment in pathology

## Abstract

**Aims:**

One of the main reliable histological features to suggest the diagnosis of inflammatory bowel disease is the presence of significant distortion of the crypt architecture indicating the chronic nature of the disease resulting in mucosal damage. This feature has a considerable intra-observer and inter-observer variability leading to significant subjectivity in colonic biopsy assessment. In this paper, we present a novel automated system to assess mucosal damage and architectural distortion in inflammatory bowel disease (IBD).

**Methods:**

The proposed system relies on advanced image understating and processing techniques to segment digitally acquired images of microscopic biopsies, then, to extract key features to quantify the crypts irregularities in shape and distribution. These features were used as inputs to an artificial intelligent classifier that, after a training phase, can carry out the assessment automatically.

**Results:**

The developed system was evaluated using 118 IBD biopsies. 116 out of 118 biopsies were correctly classified as compared to the consensus of three expert pathologists, achieving an overall precision of 98.31%.

**Conclusions:**

An automated intelligent system to quantitatively assess inflammatory bowel disease was developed. The proposed system utilized advanced image understanding techniques together with an intelligent classifier to conduct the assessment. The developed system proved to be reliable, robust, and minimizes subjectivity and inter- and intra-observer variability.

**Virtual slides:**

The virtual slide(s) for this article can be found here: http://www.diagnosticpathology.diagnomx.eu/vs/1797721309305023

## Background

The diagnosis of inflammatory bowel disease (IBD) has always been of great importance for the patient, treating physician, and pathologist. This is mainly because of the psychological, health, and social impact on the patient and the diagnostic dilemmas in some situations for the gastroenterologist and pathologists which necessitates the multidisciplinary team approach for the definitive diagnosis.

In clinical practice, the term inflammatory bowel disease (IBD) is usually reserved for ulcerative colitis (UC), Crohn’s disease (CD), or IBD with overlapping pathological features of both UC and CD resulting in a diagnosis of indeterminate colitis (IC), [1]. Both UC and CD are relapsing inflammatory conditions of gastrointestinal tract with characteristic extra-intestinal manifestations, [2]. The diagnosis of IBD, which has a considerable clinical implication for the patient with regard to treatment and prognosis, depends on the mucosal biopsies as an initial diagnostic material, [3]. Accurate diagnosis on mucosal biopsies has some limitations since the biopsies are superficial (including only mucosa) and providing little information about the depth of the disease, [2]. In many cases, UC and CD show overlapping histopathological features which are liable to inter- and intra-observer variability. Therefore, the final diagnosis of IBD requires combination of clinical, radiological, endoscopic and histological features.

The histological features of IBD include both mucosal architectural abnormalities and inflammatory features. The former is a more reliable reflection of chronicity and long standing injury and damage of the mucosa. In contrast to the normal colonic mucosa, which contains straight and tubular crypts extending to muscularis mucosa, the mucosa of IBD shows variable architectural abnormalities which are usually more prominent in UC. They include: irregular surface with pseudovillous changes, decreased crypt density and increased distance between crypts. Changes in the shape of the crypts include crypt distortion (non-parallel or cystically dilated crypts), crypt branching, crypt shortening and increased the distance between the crypt base and muscularis mucosa, [4]. Typical architectural features take several weeks to develop and maybe absent in the very early phase of IBD, [5].

The inflammatory features include diffuse transmural increase in lamina propria cellularity, neutrophil polymorph infiltration of lamina propria, cryptitis and crypt abscess, mucin depletion and paneth cell and pseudo-pyloric metaplasia, [4]. The presence of plasma cell aggregate deep in the mucosa (basal plasmacytosis) is typically seen in UC. CD, in contrast to UC, shows a more focal inflammation and a well-formed granuloma, [1].

The assessment of histological features of IBD requires the presence of full thickness mucosa containing muscularis mucosa and proper orientation of the specimen. On the other hand, the pathologist should be aware of the time of biopsy and treatment given to the patient since the histological features associated with IBD show a considerable variation with time and treatment, [5].

Quantification of pathological findings utilizing computerization and image analysis has always been a target for practicing pathologists [6-13]. The main objective of this task is to standardize the interpretation of the histological features and minimize the inter- and intra-observer variability [11,13]. There has numerous attempts in this field directed toward different pathological findings and diseases, some of which has been transformed and adopted for routine diagnostic purposes and others remain as part of research tools [6-8,10]. This science is evolving and acquiring more popularity and enthusiasm to be applied to more and more applications. We believe that this study is among the rare ones which deals with assessment biopsies for inflammatory bowel disease and would contribute to the accumulating literature and experience in this regard.

The use of standard definitions of histopathological terms makes a clear and definitive diagnosis of IBD easier, [14]. Quantitative assessment of architectural distortions through image analysis will increase the accuracy of assessment of chronicity and decrease the observer variations. The main aim of this study is to develop a computer-based system to assist the pathologist in recognizing and grading the crypt architectural distortion.

## Methods

A hundred thirty colonic biopsies were collected for examination by three pathologists who are well experienced with the required criteria for the diagnosis of IBD. These biopsies included 43 cases of normal colonic biopsies with no significant pathological changes and 87 cases of IBD with variable degrees of activity and features of chronicity including the architectural distortion mentioned above .All biopsies had at least one well-oriented 10× microscopic field representing the entire thickness of the mucosa wherever was possible.

### Study sample

The collected hundred thirty biopsies where examined by three well-experienced pathologists, independently. There was total agreement on the normal colonic biopsies. However, each expert presented his/her final assessment of the degree/grade of architectural distortion in a panel conference. It was found that the experts agreed on (50) biopsies, and had different initial opinion on the remaining (37) biopsies. An in-depth discussion was held, as a result a consensus was reached on (25) biopsies out of the (37) with a total of (75) cases. Henceforth, (12) biopsies were excluded, and only (118) biopsies were considered in the remaining of this study (43) normal biopsies and (75) IBD cases. Table 1 presents the experts consensus on the considered biopsies tabulated according to the final consensus grading. The presences of mild, moderate, and severe architectural distortions were regarded as grade I, grade II, and grade III respectively.

**Table 1:** Pathologists’ consensus grading

**Studied biopsies**	**IBD Grade**				**Excluded**
	**Normal**	**Grade I**	**Grade II**	**Grade III**	
130	43	39	26	10	12

### Image acquisition

**Figure 1:**
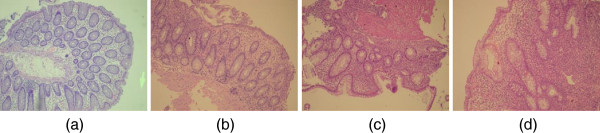
Acquired images representing IBD biopsies graded manually as (a) normal, (b) grade I, (c) grade II, and (d) grade III.

Microscopic digital colored images were captured for all 118 studied biopsies at the department of pathology, King Abdullah University Hospital (KAUH), Irbid, Jordan. Images were captured using DP20 camera set from Olympus™. DP20 is a 2 Megapixel color CCD digital microscope camera, with a resolution of 1600 × 1200 pixels, and pixel size of 4.2 μm × 4.2 μm. Images were captured at 10×, in an aim to capture the widest possible field from the tissue. The reasonably high resolution was used to obtain high quality images for processing in later stages of the assessment procedure. Furthermore, any illumination irregularities were compensated for by subtracting a background image from the captured image. The background image is captured for each slide under similar microscope setup options. Figure 1 illustrates four of the acquired images graded manually as Normal, Grade I IBD, Grade II, and Grade III, respectively.

### Image preprocessing and segmentation

Once captured and adjusted, the digital image representing a biopsy is preprocessed using image filtering and pattern segmentation techniques to locate crypts as well as the muscularis mucosa. Median image filtering and other cleaning basic operations were first used to clean up the acquired image [15]. Then, active contour model techniques were utilized to identify and localize the boundaries of crypts. “Snakes” developed by Kass *et al*., [16] is a well-known approach that is used to locate closed contours in a grayscale image. The approach and its modifications have been heavily used in many pattern recognition applications over the last two decades [16,17]. The model generates an elastic contour that is propagated by image forces towards the minimum energy generated by an image. Three forces govern the behavior of the snake; namely the internal forces, *E*_*int*_ to ensure spatial and temporal continuity, the image forces, *E*_*img*_, the main driving force of identifying the closed contour, and constraints, *E*_*con*_, which resemble application specific restrictions on the shape of the contour. An energy function is composed of the above mentioned forces. The approach propagates in an aim minimize this energy function, which in turn determines the shape of the located polygon. Interested readers might refer to [16] for further details about this technique.

### Key features extraction

As mentioned in the introduction section, the main sensitive features of chronicity in grading IBD are irregularities and/or distortion in crypts shape, the density of crypts, and the distance between the crypt base and muscularis mucosa. These features are extracted based on measurements performed on the segmented image obtained after preprocessing segmentation. To this end, the segmented image is a binary image resembling localized crypts and muscularis mucosa. These objects are identified through their boundaries as polygons in a binary image. The discriminating features are either shape related or density related. A very common approach toward quantifying shape irregularity in the boundary of a shape is the Fourier Descriptors [18,19]. Whereas, a very praiseworthy density measure is Euclidean distances calculated through Minimal Spanning Tree algorithms [20,21]. As for the distance between crypt base and muscularis, auto-regression techniques are used.

**Figure 2:**
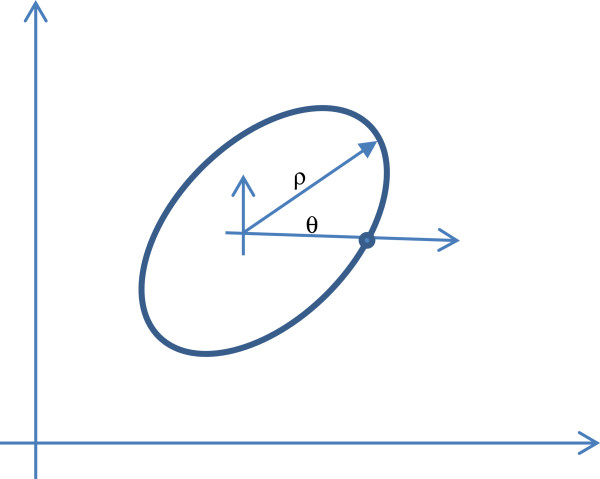
Polar coordinates representation of a boundary.

*Fourier descriptors*: are concerned with representing the boundary of a shape in parametric form. If the shape is sufficiently convex, then the boundary can be expressed in polar coordinates about some point inside the closed contour representing the shape. As can be seen in Figure 2, The boundary is specified by a function of the form *r*(*θ*), where *r* is the arc length from some point inside the closed curve, usually the centroid, to the boundary point. *r* is assumed to be single valued for all *θ* as shown in Figure 2. As can be easily seen, the parametric function, *r*(*θ*), is periodic with a period of 2*π*. Henceforth, one period is sufficient to represent the function and the Fourier Transform can be computed. Assume that *K* boundary point samples are collected to represent the curve, then the Discrete Fourier Transform (DFT) can be used to calculate the *K* complex values representing the Fourier Transform of the closed curve in the frequency domain, such that, [18]

**Figure 3:**
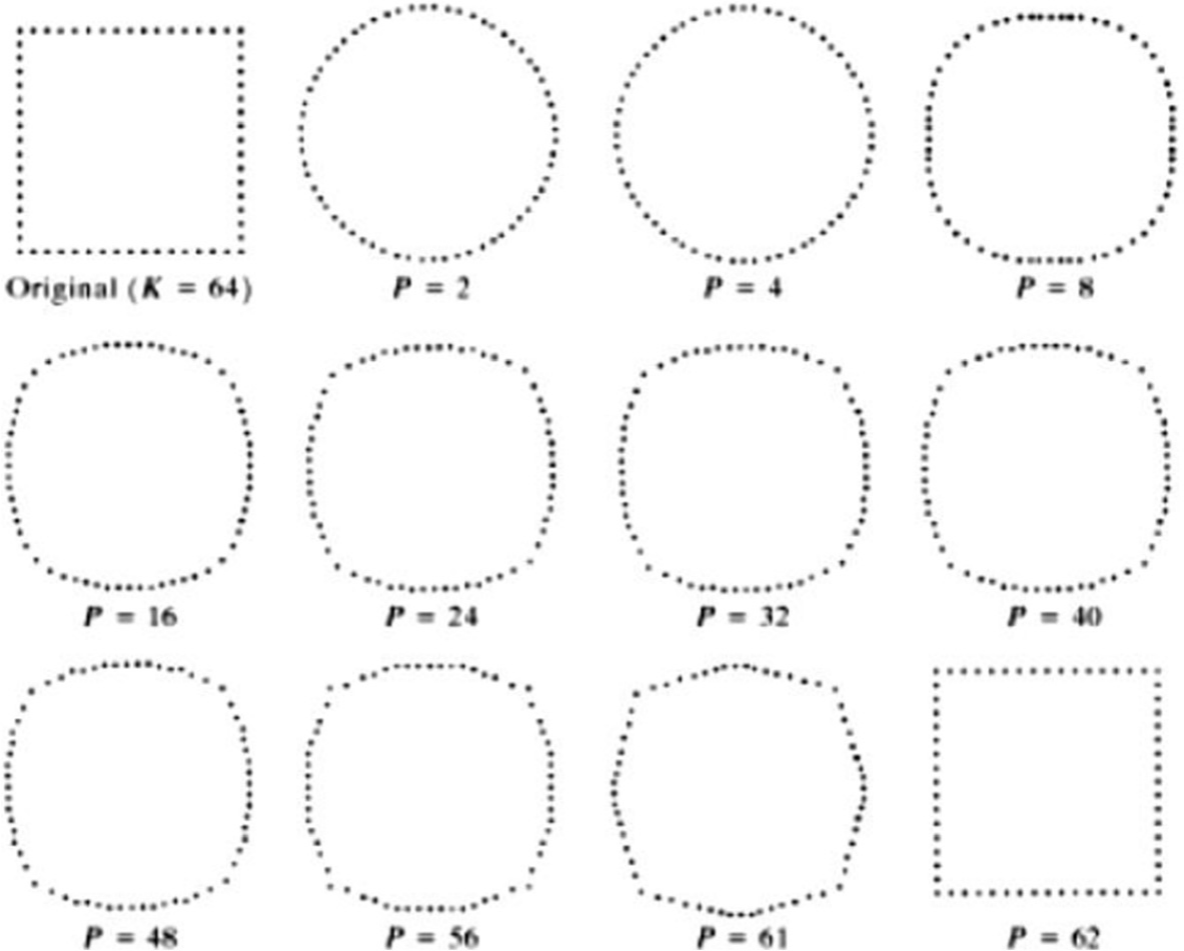
An illustrative example of shape reconstruction from Fourier Descriptors.

(1) zu=1K∑k=0K-1rke-2πukK,∀u=0,1,…,K-1,

The Fourier Descriptors are defined as the Fourier coefficients computed according to Eq. (1), and are used as the features for identification of crypts. In order to eliminate the scaling variability, the Fourier Descriptors should be normalized. The position invariance is obtained by nullifying the 0^th^ order Fourier Descriptor. Size invariance is obtained by dividing all Fourier Descriptors by the magnitude of the 1^st^ order Fourier Descriptor. Rotation invariance is obtained by considering only the magnitude of the Fourier Descriptors and neglecting the phase information. Since crypts are normally smooth and tend to get distorted due to inflammatory cells, then most information is contained in low order coefficients. For that, only the first to ninth coefficients are considered hereafter after nulling the 0^th^ order coefficient. Figure 3 shows an illustrative reconstruction example of a shape using the Fourier Descriptors.

#### ***Minimal spanning tree***

Given a set of points or vertices in the space, the Minimum Spanning Tree (MST) problem calls for finding a sub-tree spanning all the vertices whose total weight is minimal. It is conjectured by any computer scientists that this problem has deterministic linear time complexity. Many algorithms have been developed in the past years to deduce the minimal spanning tree of a connected weighted graph [20,21]. Kruskal’s algorithm is an example of a greedy algorithm that is commonly used in many graph theory applications [20]. It starts by creating a forest (set of trees), where each vertex in the graph is a separate tree. Then, it creates a set S containing all the edges in the graph. Based on this set S, and while not empty, it propagates by removing an edge with minimum weight from S, if the edge connects two different trees, then it adds this edge to the forest combining two trees into a single tree, otherwise it is discarded. The algorithm keeps iterating until the forest reduces to one tree. This tree is called the minimum spanning tree (MST). This algorithm was adopted and implemented such that, the set of vertices represent the centroids of located crypts in an image. The coordinates of the centroid are defined as

(2) $$\begin{array}{cc} \bar{x}=\frac{M_{10}}{M_{00}} & \bar{y}=\frac{M_{01}}{M_{00}}, \end{array}$$

where *M*_*pq*_ is the *p*^th^*q*^*th*^ order moment of a function *f*(*x*,*y*) and is defined as

(3) $$M_{\mathrm{pq}}=\int_{-}\int_{\infty }^{\infty }x^{p}y^{q}f\left(x,y\right)\begin{array}{cc} \mathrm{dx} & \mathrm{dy} \end{array}$$

Once calculated, the MST information is used to calculate the average spacing distance between crypts’ centroids in an image as a discriminating feature for further analysis in later stages of the process. This distance is normalized by dividing the computed average by the maximum distance encountered.

#### ***Auto-regression***

Given a set of points in the space, a first order polynomial could be used to fit the data of the form ( *y* = a*x* + b), where a is the slope and b is the y-intercept. Both a and b can be found by minimizing the mean square error (MSE) defined as

(4) $$\text{MSE}={\sum \left(y_{i}-\left(ax_{i}-b\right)\right)}^{2},$$

This approach is used to fit two first order curves. The first represents the muscularis through the set of boundary points located using the “snakes” algorithm. The second curve is drawn based on the closest point of the first line of crypts encountered. Once the two lines are deduced and computed. The average Euclidean distance between the two curves is measured. Norms are computed starting from every point of the muscularis curve ending at the line representing the crypts throughout the image plane. Then the average distance is calculated. This measure is divided by maximum distance encountered for normalization purposes. The normalized average distance is then used as a discriminating feature hereafter.

### Classification

**Figure 4:**
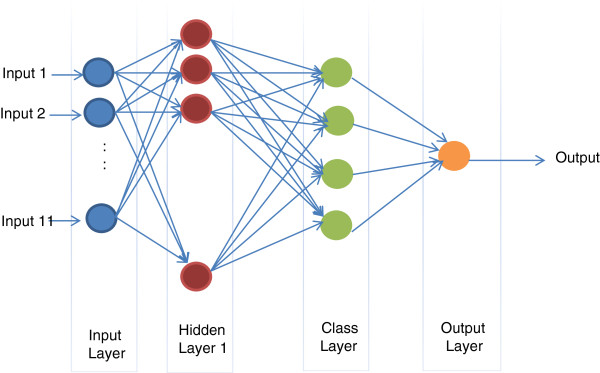
Classifier Architecture.

The colonic biopsies included in this study are classified into one of the four categories; namely normal, mild, moderate, and severe architectural distortion. In order to automatically conduct the assessment, an intelligent classifier is needed. Traditional statistical classification procedures such as discriminant analysis are built on the Bayesian decision theory [22]. In these procedures, an underlying probability model must be assumed in order to calculate the posterior probability upon which the classification decision is made. One major limitation of the statistical models is that they work well only when the underlying assumptions are satisfied. Artificial Neural Networks (ANN) are commonly used as an alternative classifier. In particular, Probabilistic Neural Network (PNN) is mainly specialized for use with classification problems [23,24]. The architecture of the PNN is an implementation of the Bayesian classifier in which a feature vector, $\vec{V}$, is assigned to a class *C*_*i*_, if and only if

(5) $$\begin{array}{cc} p_{i}L_{i}f_{i}\left(\vec{V}\right)>p_{j}L_{j}f_{j}\left(\vec{V}\right) & i\neq j \end{array}$$

where *p*_*i*_ is the probability that $\vec{V}$ belongs to class *C*_*i*_. Li is the loss function associated with misclassification, and *f*_*i*_($\vec{V}$) is the probability density function (pdf) for class *C*_*i*_. The Archeticture of a PNN is similar to a four-layered feed neural network. The first layer, called the input layer, consists of as many nodes as the inputs, which is eleven in this case. The inputs should be normalized before being processed. The second layer, called the hidden layer, is fully connected with the input layer. The number of nodes is similar to the number of training patterns used. The activation function is of exponential form [24]. The third layer, called the class layer, accumulates the outputs from the hidden layer nodes that belong to the same class. It consists of as many nodes as the number of classes, which are four in our case. Finally, the forth layer, called the decision layer, calculates the final output according to Eq (5), which determines the classified class. The classifier architecture is shown in Figure 4. Interested reader can refer to [23,24] for further theoretical details about PNN.

**Figure 5:**
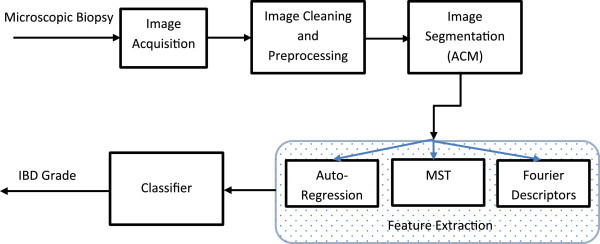
Flow of the proposed methodology.

To summarize, the developed methodology starts by acquiring a digital image representing the biopsy under study. The acquired image is them preprocessed for cleaning purposes, then “snakes” algorithm is used to locate closed contours representing crypts. For each encountered crypt, the first ten Fourier Descriptors are computed and normalized as described above. Average Fourier Descriptors are then calculated for each image. These averages are used as discriminating shape related features. On the other hand, the centroid for each localized crypt is computed, based on that, the minimal spanning tree is deduced, and the average distance between crypts is calculated and normalized accordingly. This distance is used as a density related discriminating feature. Finally, the distance between muscularis and base crypt line is calculated and normalized. This measure is then used as a discriminating feature of crypts loss. All eleven (11) normalized features are then fed to the classifier (PNN) to conduct the assessment, Figure 5.

## Results and discussion

**Figure 6:**
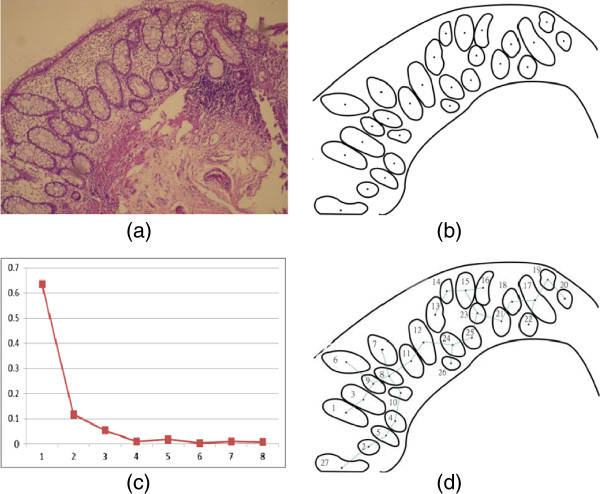
**An illustrative example. (a)** original image, **(b)** image after segmentation and localizing the crypts, **(c)** the corresponding FDs of, and **(d)** the corresponding arcs after running the MST algorithm.

A database representing the 118 cases was developed, with each tuple in the database resembles the acquired image, and the corresponding eleven extracted features. The eleven features were extracted for each acquired image according to the developed methodology discussed in the previous section. Figure 6 shows an illustrative example, which portrays the processing steps of the proposed methodology. The figure shows the original image, followed by the binary image after applying preprocessing filtering and cleaning followed by the segmentation process using the developed snakes-like algorithm to localize the crypts. After that, the segmented binary image is used to extract the key features; namely the Fourier Descriptors (FDs), Figure 6-c, and the MST arcs calculated based on the adopted MST algorithm, Figure 6-d. This information is used to deduce and calculate the eleven key features.

**Figure 7:**
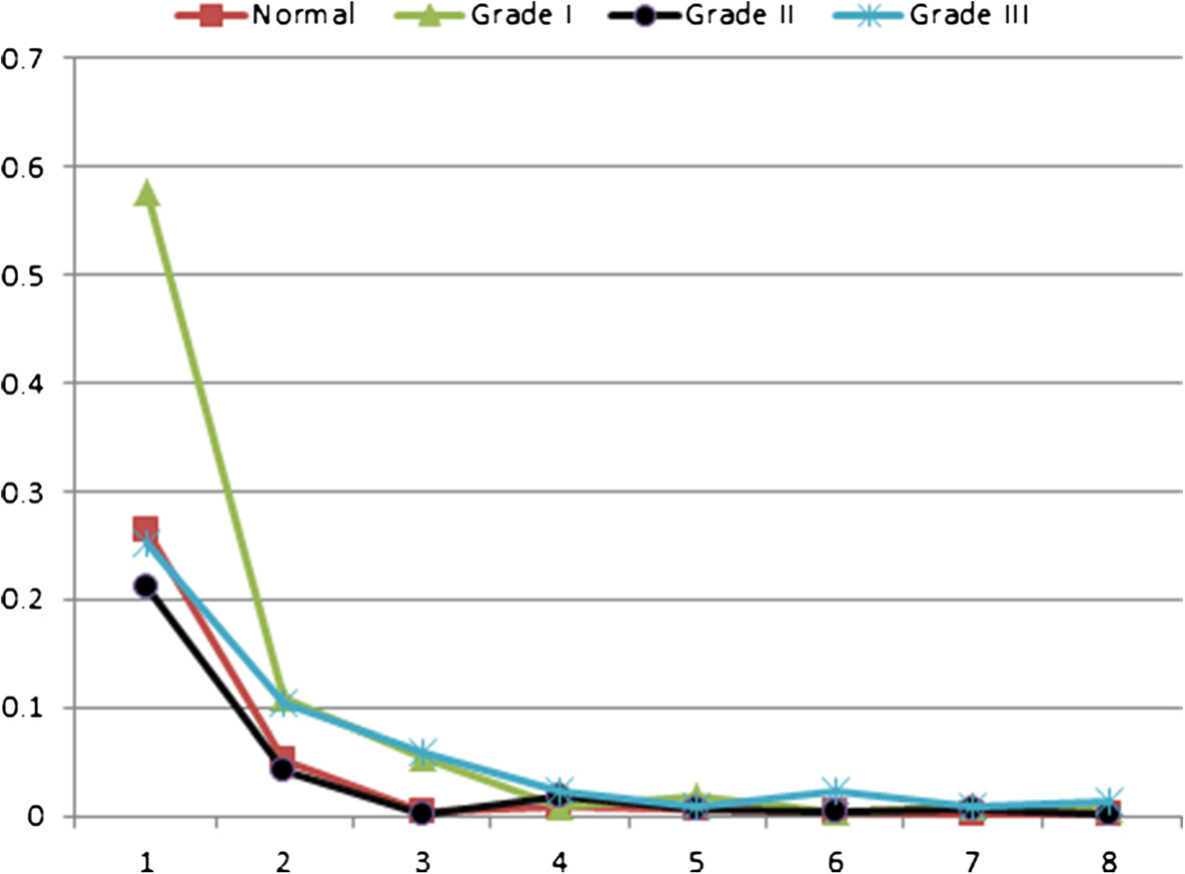
**The 1**^**st **^**to 9**^**th **^**order Fourier Descriptors of four cases graded manually as normal, grade I IBD, grade II, and grade III.**

**Figure 8:**
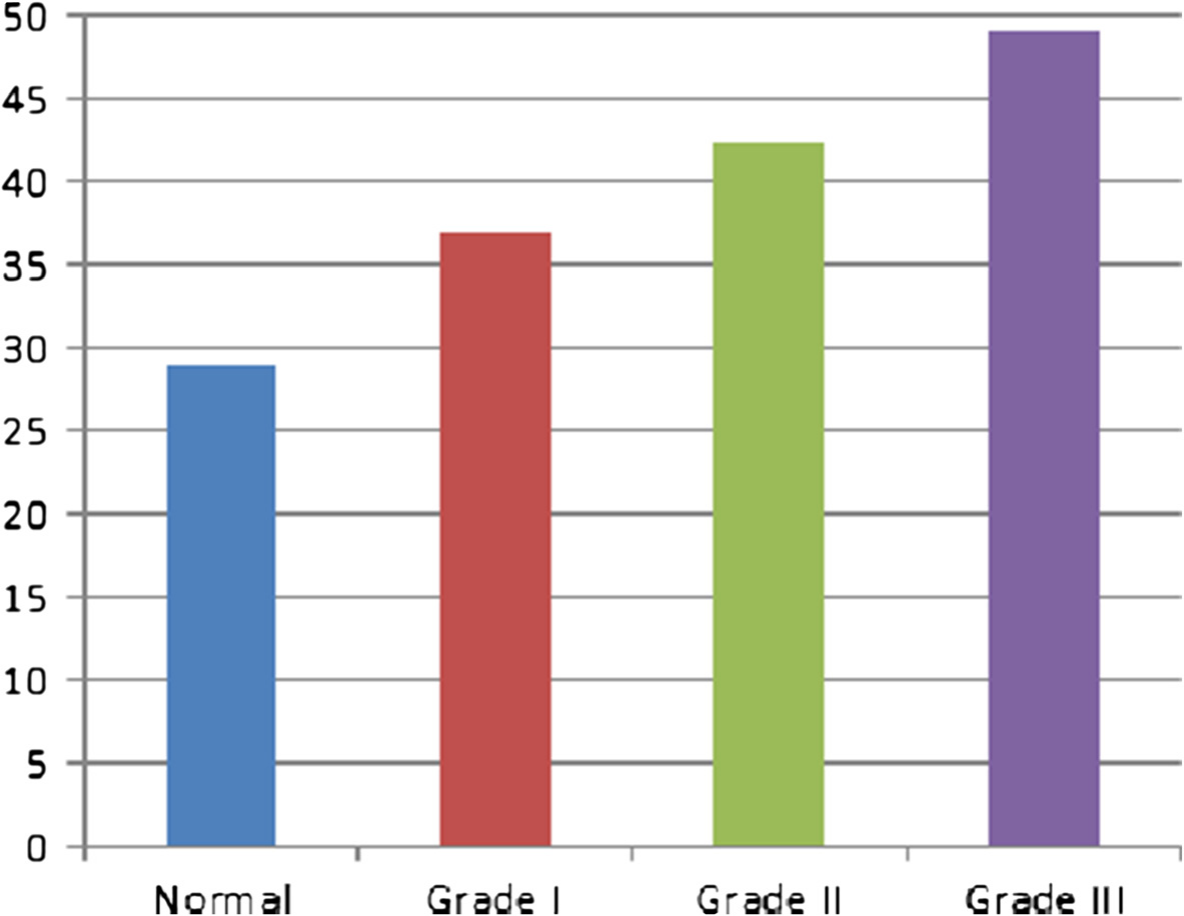
The average crypts spacing (density) measured using minimal spanning tree.

**Figure 9:**
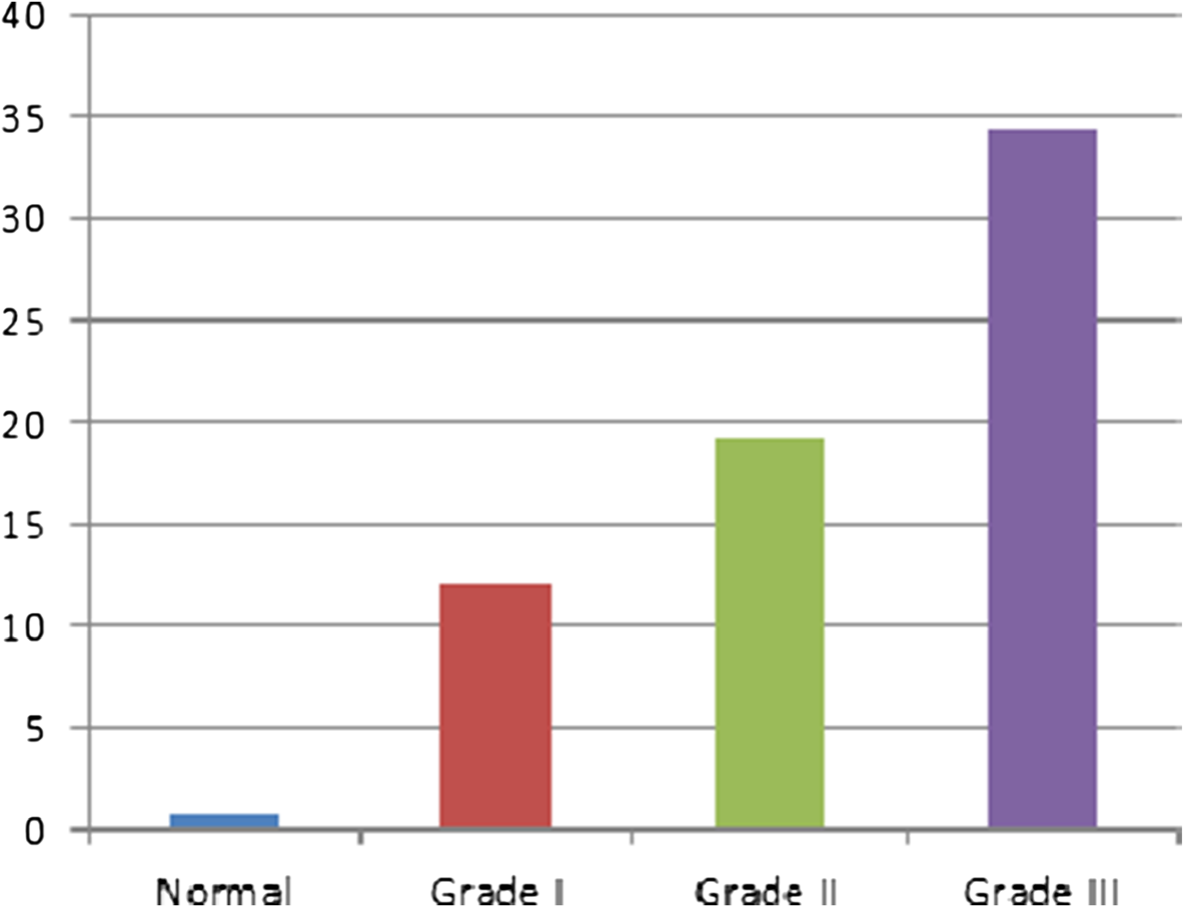
Average distance between muscularis mucosa and the first line of crypts calculated using auto-regression techniques.

For further illustration, four typical cases graded manually as normal, IBD grade I (Mild), grade II (Moderate), and grade III (Severe) were processed according to the proposed methodology. The obtained features are presented in Figures 7, 8 and 9. Figure 7 shows the 1^st^ to 9^th^ order Fourier Descriptors (FDs) of the four cases. Figure 8 shows the average crypts density calculated based on minimal spanning tree (MST). Finally, Figure 9 shows the average distance between muscularis mucosa and the first line of crypts calculated using auto-regression techniques. In this way, all biopsies considered in this study were processed and the database is constructed.

The final experts’ consensus was used as a ground-truth for the classifier (PNN) during the training and testing phases. Two thirds of the cases (79 biopsies) were used for training purposes and one third (39 biopsies) for testing. The nature of the classifier requires training to adapt its parameters to reach the optimal setting to meet the fed diagnosis. While during the testing, the system works blindly and the diagnosis is used for system evaluation purposes only. *Precision* and *Recall*, are used to evaluate the developed system in contrast with experts’ consensus. According to [25], *Precision* is defined as the ratio of correctly classified cases to the total number of correctly and incorrectly classified cases, whereas, *Recall* is defined as the ratio of the correctly classified cases to the total number of studied cases in a certain grade. Table 2, shows the system performance during the training phase. As can be seen from the table, the classifier managed to adapt very well and achieved a 100% recognition rate during the training phase.

**Table 2:** System performance during the training phase in contrast with expert’s consensus

**Experts’ consensus**		**System classification**			
**IBD grade**	**Cases**	**Normal**	**IBD**	**IBD**	**IBD**
			**Grade I**	**Grade II**	**Grade III**
Normal	29	29	0	0	0
I	26	0	26	0	0
II	17	0	0	17	0
III	7	0	0	0	7
Precision		100%	100%	100%	100%
Recall		100%	100%	100%	100%

Once set, the system is utilized to test the remaining cases using the optimal parameters obtained during training. While the system was fed with the experts’ consensus during the training phase, it works independently and blindly during the testing phase. Table 3 portrays the results obtained during this phase.

**Table 3:** System performance during the testing phase in contrast with expert’s consensus

**Experts’ consensus**		**System classification**			
**IBD grade**	**Cases**	**Normal**	**IBD**	**IBD**	**IBD**
			**Grade I**	**Grade II**	**Grade III**
Normal	14	13	1	0	0
I	13	0	12	1	0
II	9	0	0	9	0
III	3	0	0	0	3
Precision		100%	92.31%	90%	100%
Recall		92.86%	92.31%	100%	100%

The overall system performance during training and testing phases is illustrated in Table 4.

**Table 4:** Overall system performance in contrast with expert’s consensus

**IBD grade**	**Performance measure**	
	**Precision**	**Recall**
Normal	100%	97.67%
Grade I	97.44%	97.44%
Grade II	96.30%	100%
Grade III	100%	100%
**Overall**	**98.31%**	**98.31%**

As can be recited from Tables 2, 3 and 4, the proposed system was able to correctly classify 116 biopsies out of 118, with an overall precision of 98.31% as compared with experts’ consensus. This sets great confidence in the proposed system in producing highly accurate assessment of architectural distortion and mucosal damage as an evidence of IBD diagnosis and extraordinary reproducibility. Further investigation of the misclassified cases, we found that those cases where among the cases experts had different opinions on in their first round, and only after panel discussion, they reached a consensus. This also emphasizes the well-recognized difficulty of an initial diagnosis of IBD in its early phase with only subtle distortion of the crypt architecture.

As far as authors are aware, the proposed system is the first attempt ever accomplished to automate assessment of crypt architecture distortion and mucosal damage in the diagnoses process of inflammatory bowel disease. The selected set of features to quantify the irregularities in crypts shape and distribution was the key step in the automation process. We strongly believe that the proposed system can be the core for automated and quantitative assessment of IBD, which in turn, can reduce dramatically inter- and intra-observer variability, and can achieve high reproducibility.

## Conclusions

In this paper, a novel automated system was developed to quantitatively assess mucosal damage and architectural distortion in inflammatory bowel disease (IBD). The proposed system relies on advanced image understating and processing techniques to segment digitally acquired images of microscopic biopsies, then, to extract key features to quantify the crypts irregularities in shape and distribution. These features were used as inputs to an artificial intelligent classifier that, after a training phase, can carry out the assessment automatically. The developed system was evaluated using 118 IBD biopsies. 116 out of 118 biopsies were correctly classified as compared to the consensus of three expert pathologists, achieving an overall precision of 98.31%. The proposed system is very reliable, robust, and minimizes subjectivity and inter- and intra-observer variability. We strongly recommend this proposed system to be used by practitioners and researchers in this field. The relationship between the degree of mucosal damage and architectural distortion in the initial presentation and the degree of response to treatment as well as risk of developing dysplasia and subsequent carcinoma is another venue for future studies.

## Competing interests

The authors declare that they have no competing interests.

## Authors’ contributions

IIM, is the principal researcher and he conducted the collection of cases and studied them manually, performed the grading, and evaluated the proposed system together with Al-Omari FA. FAA-O, is the computer specialist who together with the first autoht decided on the key features describing the 4 grades of IBD. Then he was in charge of designing and implementing the proposed system, performing the evaluation of the proposed system and discussing the results with other authors. RMS, and AHM were in charge of preparing the biopsies, grade them manually and together with the first author hold a panel to reach consensus. All authors participated in preparing the manuscript according to their specialty and interest. All authors read and approved the final manuscript.
